# The Pharynx

**Published:** 1917-02

**Authors:** John C, Warbrick

**Affiliations:** Chicago


### The Pharynx.

By JOHN C, WARBRICK, M. D., Chicago.

The pharynx extends from the base of the skull to the lower border of the cricoid cartilage opposite the sixth cerincal vertebra, being from about four and a half to five inches long.

Just where the pharynx is situated and what it is may be better understood by giving its numerous attachments.

It may be said it is situated at the back of the nose and tongue in front of the upper part of the spinal column and at the sides behind the posterior pillers of the fauces.

If the comparison can be made the pharynx may be described as somewhat resembling an empty bag of flour, pulled out at the top in several places and narrowed a little towards the bottom.

It walls in and connects closely together all the parts extending from the base of the skull to the opening of the esophagus, thus serving as a kind of a bag to allow the food to pass down into the esophagus. It has the following numerous attachments: To the base of the sphenoid bone and basilar process of the occipital bone; to petrous part of temporal bone; to posterior nares; to lower jaw; to mouth; to larynx; to styloid process of hyoid bone; to the six upper cervical vertebrae; to the posterior maxillary ligament.

Anteriorly it is incomplete, the space being occupied by the cavities of the nose, the mouth and larynx, also the thyroid and cricoid cartilages.

Posteriorly it is connected by loose areolar tissue to the cervical portion of the vertebral column, the longi colli muscles and the recti capitis antici.

Laterally it is connected to the styloid process and muscles.

When at rest the anterior and posterior walls of the pharynx are supposed to be closer together. It communicates with seven openings as follows: Two eustachian tubes; two posterior nares, the mouth, larynx and esophagus. It is wider from side than from before back. Its widest part is at the level of tip of greater corun of the hyoid bone about 2 inches. Its narrowest part is at the cricoid cartilage where it ends in the esophagus at which place the trachea begins.

A number of important structures are in close relationship to the sides of the pharynx on the outside.

The internal carotid artery is so close its pulsations may be felt through the mouth by the finger.

The common carotid artery is also closely related to it, as well as the internal jugular veins.

The following nerves are also structures, not far away: glosso pharyngeal, spinal accessory, pneumogastric, sympathetic and hypoglossal.

When a person hawks up mucous or scrapes the throat the material comes from the posterior wall of the pharynx, for when one makes an examination mucous in small pieces or string-like may often be seen on either side of the wall, which is more marked in some cases than in others. This is what comes away when the throat is scraped to get rid of it from time to time, when it collects as it does and often causes a tickling in the throat. There is a condition that is called by the peculiar term rheumatic throat in some individuals which may be more or less common.

This is a term no doubt given to chronic pharyngitis, and it is not a good one to use on account of being vague and indefinite. A rheumatic throat means that there is a thickening of the tissue on the posterior wall of the pharynx in one or more places. This tissue becomes fibrous and tied down so that it may be seen as whitish spots on the pharyngeal wall.

It would seem to come from a chronic state and to affect those in middle life or later years.

---

**Destruction of House Flies.** Earle B. Phelps and Albert F. Stevenson, Pub. Health Reports, Nov. 3, consider formaldehyd and sodium salicylate as the best. Use 15 c.c. of either the 40% solution of formaldehyd or the powdered sodium salicylate to 500 c.c. of water. Nearly fill a tumbler, cover it with a circular piece of blotting paper and invert a saucer over the glass. Turn the whole combination upside-down and insert a bit of match or tooth-pick to allow entrance of air. A little sugar sprinkled over the paper will add to its attraction for flies. These solutions are practically harmless in the quantities liable to be drunk by children and domestic animals.

---

**Treatment of Syphilis with Luargol, or 102.** (St. Paul Med. Jour., Edit., Nov.) Danysz of the Pasteur Institute of Paris has elaborated dioxy-diamino-arso-benzol-stibico-silver sulphate, whose short name and number are as above. 5-10 c.b. doses are efficient, while the toxicity is 0.20 per kilo of rabbit. Injections are given intravenously, repeated every 2 or 3 days. Ordinarily, the first dose is 0.10-.15, increasing to .35. The total dosage required is 1.-1.50. The solutions are easily prepared by dilution of concentrated solutions and administered by 20 c.c. glass syringes, in the office, the patient requiring no after precautions.

